# Beyond CPAP: modifying upper airway output for the treatment of OSA

**DOI:** 10.3389/fneur.2023.1202271

**Published:** 2023-07-21

**Authors:** Eli Gruenberg, Jessica Cooper, Tania Zamora, Carl Stepnowsky, Andrew M. Vahabzadeh-Hagh, Atul Malhotra, Brandon Nokes

**Affiliations:** ^1^Division of Pulmonary, Critical Care, and Sleep Medicine, University of California, San Diego, La Jolla, CA, United States; ^2^Health Services Research and Development, Veteran's Affairs (VA) San Diego Healthcare System, San Diego, CA, United States; ^3^Department of Otolaryngology—Head and Neck Surgery, University of California, San Diego, La Jolla, CA, United States; ^4^Sleep Section at the Veteran's Affairs (VA) San Diego Healthcare System, San Diego, CA, United States

**Keywords:** genioglossus, hypoglossal nerve, precision medicine, control of breathing, sleep disordered breathing, positive airway pressure

## Abstract

Obstructive Sleep Apnea (OSA) is exceedingly common but often under-treated. Continuous positive airway pressure (CPAP) has long been considered the gold standard of OSA therapy. Limitations to CPAP therapy include adherence and availability. The 2021 global CPAP shortage highlighted the need to tailor patient treatments beyond CPAP alone. Common CPAP alternative approaches include positional therapy, mandibular advancement devices, and upper airway surgery. Upper airway training consists of a variety of therapies, including exercise regimens, external neuromuscular electrical stimulation, and woodwind instruments. More invasive approaches include hypoglossal nerve stimulation devices. This review will focus on the approaches for modifying upper airway muscle behavior as a therapeutic modality in OSA.

## Introduction

### OSA overview and underlying pathogenic mechanisms

Obstructive sleep apnea (OSA) is a common and heterogeneous condition that affects up to one billion individuals globally (1). OSA left untreated is associated with severe comorbidities, including diabetes mellitus (2), coronary artery disease (3), increased risk of stroke (4), congestive heart failure (5), atrial fibrillation (6), and possibly death (7). While continuous positive airway pressure (CPAP) is the gold standard, adherence is highly variable (8). The 2021 global CPAP shortage highlighted the need for different approaches to OSA management (9). Conventional approaches to those who are CPAP intolerant include positional therapy, weight loss, oral appliances, and upper airway surgery (10). Our lab and others are attempting to understand the pathophysiological drivers of OSA to personalize therapeutic options (11). The OSA traits (endotypes) will not be reviewed extensively here but include: (1) excessively collapsible upper airways, (2) inadequate muscle compensation, (3) ventilatory control instability (high loop gain), and (4) low respiratory arousal threshold (ArTH) (12). This review will focus on studied modalities for improving upper airway dilation as potential OSA treatments. We will examine the role of upper airway training and electrical stimulation of the upper airway muscles and nerves as therapeutic options for OSA (13). Notably, drug therapy for improving upper airway motor output is also an active area of investigation but is beyond the scope of this review (14–16).

### Overview of the respiratory upper airway

The upper airway consists of 23 pairs of muscles, including dilators, protrudors, retractors, and the intrinsic muscles of the tongue (17, 18). These muscles are state-dependent, meaning that their activity level tends to decrease with sleep onset (19), especially with rapid-eye movement (REM) sleep (20, 21). Concerning OSA pathogenesis, genioglossus is the best studied of these muscles due to its ease of access [i.e., with electromyography (EMG) wires] (22).

However, multiple upper airway dilators and constrictors are important in the upper airway response to flow-limited breathing during sleep (23). Indeed, the superior, middle, and inferior pharyngeal constrictor muscles constrict and decrease airway caliber during times of increased airway volume (such as during inspiration), but have dilatory action when airway volumes are low (such as at the end of an apnea) (23). The pharyngeal retractors styloglossus and hyoglossus, while typically known for decreasing airway caliber on their own, may have a synchronous effect with genioglossus to promote upper airway patency (24). The peripharyngeal muscles as well as the intrinsic muscles of the tongue are also important in maintaining luminal patency amidst flow limitation (25, 26). Additionally, the muscles of the soft palate palatoglossus, palatopharyngeus, levator palatini, tensor palatini in addition to other muscle groups are important in combatting obstructive events of the upper airway (27).

Upon sleep onset, the upper airway relies on chemoreceptive cues, mechanical load, and lung volume afferent cues to drive firing patterns for each breath cycle (22). There is a negative pressure reflex, in which inspiratory negative pressure across the upper airway increases genioglossus output (28). This reflex is generally attenuated during sleep compared to wakefulness, but is augmented during supine sleep vs. recumbent (28, 29). Both mechanical loading and elevated pCO_2_ increase upper airway dilator output, with an additive effect when these two stimuli are combined (22). In many cases of OSA however, the efficacy of upper airway dilators in maintaining pharyngeal patency is reduced (30). This loss of efficacy is partly related to a decrease in the state-dependent drive but also may emerge from an inadequate muscle output to compensate for an excessively collapsible upper airway (20). The importance of upper airway neuromyopathy has been debated, with data somewhat mixed regarding whether observed abnormalities are a cause or consequence of disease (31–35). There may also be muscle asynchrony contributing to the loss of pharyngeal patency in sleep (36). With consideration of the role of upper airway muscle function in sleep apnea pathogenesis, a number of strategies have been undertaken to improve upper airway performance in response to flow-limited breathing.

### Attempts at improving muscular dilation of the upper airway

#### Myofunctional therapy for the treatment of OSA

While the mechanisms of OSA pathogenesis are heterogeneous, exercises for improving upper airway stability through muscle training and improvement in passive pharyngeal properties [such as the critical closing pressure (PCrit)] have been pursued in clinical research (37). The ideal training regimen, training method, and patient selection for improving OSA is yet to be determined. Still, there may be an improvement in sleep apnea severity, and daytime symptoms with dedicated upper airway training regimens often referred to as myofunctional therapy (MT), though the data is inconsistent (38). MT has been predominantly studied in mild to moderate OSA (39). The exercises prescribed are heterogeneous and the relative mechanisms for these exercises to combat OSA are uncertain. There have also been studies of MT in severe OSA, where MT appears less effective but may serve as a CPAP adjunct (40). Exercises are reported to target the soft palate, tongue, and external facial muscles (38).

A common combination of the above exercises is appended below (Table 1). Exercises are typically intensified over the course of a 6-week training period.

**Table 1:** Representative MT regimen prescribed to patients with mild-moderate OSA.

**Category**		**Exercise name**	**# seconds**	**# repititions**	**# sessions/day**
Tongue	1	Tongue Press	5	5x	2
	2	Stick Your Tongue Out	5	5x	2
	3	Stick Your Tongue Out and Down	5	5x	2
	4	Stick Your Tongue Out and Up	5	5x	2
Soft palate	1	Blowing with Resistance with Balloon	5	10x	2
	2	Say “Ahhh”	10	10x	2
Throat and neck	1	Ceiling Swallow	5	10x	2
	2	Going Up	10	10x	2
Jaw and lips	1	Lip Workout	10	10x	2
	2	Jaw Resist	10	10x	2
	3	Chewing			

The regimen advances and is modified over a 6-week period. Adapted from Guimaraes et al. (37). This is solely meant for illustrative purposes, and the ideal MT training regimen is unclear.

#### Benefits and limitations of myofunctional therapy

In some randomized controlled trials (RCTs), MT demonstrated improvements in polysomnographic measures of sleep, including AHI and oxygen saturation parameters (10). In a meta-analysis including observational studies, MT elicited a 50% decline in the AHI among adults and a 62% decline in the AHI among children (38). MT also demonstrated improvements in secondary outcomes, including subjective quality of life scores, Epworth Sleepiness Scale (ESS), snoring, and CPAP compliance (38). The mechanism(s) of MT on AHI reduction are heterogeneous and not fully delineated (10, 37). Notably, MT has also been used as an adjunct to improve CPAP adherence (41). However, a major limitation of MT is the lack of standardization. Generalizability between MT studies remains low due to variable inclusion criteria, follow-up protocols, exercise regimens, and training devices (10). Additionally, the mild severity of OSA within the available studies creates the possibility of regression to the mean explaining some of the positive reported results for MT. The ideal anatomy for MT benefit, i.e., based on Mallampati/Friedman scores, for instance, is unclear. The durability of effect of MT is also uncertain (39). Barriers to adherence with MT are potentially related to lack of patient engagement/understanding once they are in a home setting and practicing MT exercises independently (42). According to the European Respiratory Society guidelines, MT is not recommended as a treatment unless patients are reluctant to engage in surgical/mechanical strategies (43). Further research on MT should focus on determining which exercises yield maximal benefit, which patients benefit from MT, and which therapeutic adjuncts can and should be added for an individual based on their unique OSA traits.

According to the European Respiratory Society guidelines, MT is not suggested as a standard treatment for OSA [43]. The guidelines recommend patients use CPAP instead of MT (43). However, patients who are reluctant to engage in surgical/mechanical strategies may find improvements in their symptoms (43). These recommendations are conditional and are based off a low quality of evidence. More research on MT is necessary to provide confident recommendations.

### Upper airway training with woodwind instruments

Over the past 20 years, it has been noted that woodwind instrument playing may have a protective effect on OSA (44). In 2006, Puhan noted that playing the didgeridoo, an indigenous Australian instrument, improves the AHI compared to controls (44). This study prompted the investigation of other woodwind instruments for treating and preventing OSA (45). In a study comparing wind instrument musicians to string instrument musicians, no significant differences in sleep efficiency or subjective sleep quality metrics were noted (46).

### Didgeridoo

The use of woodwind instruments such as the didgeridoo may be beneficial in the treatment of symptomatic OSA. In a study by Puhan and colleagues, didgeridoo practice showed significant improvement in AHI, ESS, and partner sleep disturbance scores (44). In a meta-analysis of the effects of musical interventions in OSA, the didgeridoo was the most therapeutic musical intervention in improving sleep-disordered breathing (45). This finding may be due to the unique nature of the didgeridoo requiring circular breathing (45). Circular breathing is the vocalization of a continuous tone while simultaneously inspiring through the nose. This procedure is performed by expelling air through the mouth and using the cheek muscles to create a reservoir of air. Notably, however, in other instruments requiring circular breathing, such as the bassoon, circular breathing in and of itself has yet to be shown to be effective in treating OSA consistently (47).

Puhan and colleagues, are the only research group to research the effects of the didgeridoo on OSA thus far to our knowledge (44). One major limitation of this study was the small sample size of 25 participants and the lack of a rigorous control group. The control group consisted of participants put on a waiting list. This approach was viewed as easier than having participants practice with a “sham” didgeridoo. A clear role of didgeridoo playing in OSA treatment is not defined (48).

### Other woodwind instruments

Subsequent studies have separated instruments into single-reed, double-reed, high-brass, and low-brass instruments (48). Single reed instruments (clarinet, saxophone) include a single piece of cane that vibrates when sound is introduced. In contrast, double reed instruments (bassoon, oboe, English horn) have two pieces of cane that vibrate and a narrower aperture. Low brass includes tubas and sousaphones. High brass includes trumpets and French horns. Of the instruments noted, the double reed appears to improve AHI and daytime symptoms consistently, with more hours spent playing corresponding to greater AHI reduction (48). Ward et al. argued that the narrower aperture of double reed instruments and requisite air pressure were comparable to high-brass instruments (30–42 mmHg vs. 13–42 cmH_2_O) and thus did not explain the differences in efficacy across the woodwinds. Additionally, benefit in OSA treatment was not seen in non-wind instrumentalists (48). Rather, they speculated that the differences in efficacy were attributable to the differences in muscle activation patterns across the instruments (48). Circular breathing does not have a clear and consistent role in improving the AHI (47). Although, the extent of circular breathing and requisite practice requirement of the didgeridoo may be greater than in other instruments and thus involve a more intensive circular breathing practice (48). While woodwind instruments may be helpful for sleep apnea, which instruments to use and how to implement them remains uncertain (49).

#### Electrical stimulation of the upper airway

**Figure 1:**
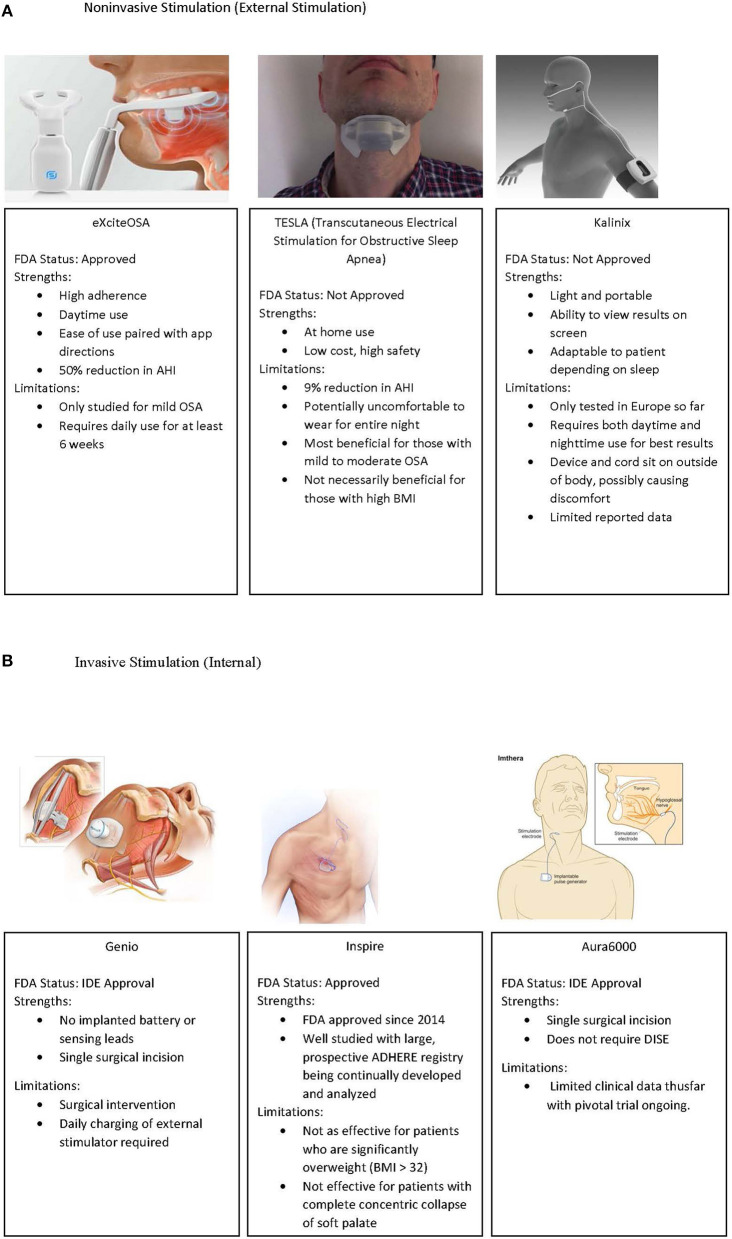
**(A)** External stimulation devices which have been utilized in OSA treatment. Left to right, ExciteOSA, TESLA, and Kalinix devices. **(B)** Implantable upper airway stimulation devices, including Genio, Inspire, and the Aura6000 device. IDE, Investigational Device Exemption. Image re-use permissions granted where applicable (51, 86, 87).

Electrical stimulation of the upper airway has included both external stimulation of upper airway muscles and direct stimulation of nerves supplying the upper airway. Current devices for external and internal (surgical) stimulation of the upper airway muscles and nerves, respectively, are shown in Figure 1.

### External submental electrical stimulation

External stimulation of the upper airway dilator muscles has recently become a clinically significant modality in treating mild OSA. Devices like eXcite OSA and TESLA offer symptom relief for primary snoring and OSA (50, 51). External stimulation of upper airway muscles has come about through various approaches, predominantly focused on nighttime tongue stimulation.

The initial attempts at electrically stimulating the upper airway during flow-limited breathing were by Miki et al. (52). Using submental electrodes and a microphone over the cervical trachea, electrical stimulation of 15–40 V at a frequency of 50 Hz was applied when tracheal breath sounds were < 15% of tracheal sounds during tidal breathing for 5 s (52). This study was in six patients and showed decreased sleep apnea severity and increased stage III sleep without associated arousals (52). This same group showed that direct stimulation of genioglossus in anesthetized dogs decreased upper airway resistance (53). Hillarp et al. later used submental electrical stimulation in a single patient during apneic events. The behavior of the upper airway was recorded using videoradiography and showed that tongue base obstruction improved with submental stimulation (54).

Edmonds et al. subsequently used a transcutaneous neuromuscular stimulation device (TENS) to assess the efficacy of concurrent submental and infrahyoid stimulation on OSA severity. No significant reduction in AHI was noted (55). Additional efforts entailing multi-site stimulation emerged in the following years. Guilleminault attempted simultaneous submental and transmucosal sublingual stimulation with a proprietary device without significant change in OSA parameters (56). Schnall also attempted simultaneous submental, paralaryngeal, and submucosal stimulation using a horseshoe shaped electrode while measuring pharyngeal resistance as the primary outcome measure. Only sublingual resistance improved (57).

In 1999, Wiltfang et al. applied daytime submandibular electrical stimulation to suprahyoid muscles by intra and extraoral electrodes via a transcutaneous electrical nerve stimulation (TENS) unit. After a 4-week training regimen (30 min twice a day), the researchers documented suprahyoid hypertrophy by ultrasound, reduced respiratory disturbance index from 13.2 to 3.9/h, and reduced oxygen desaturation index from 23 to 2.8/h. Despite these improvements, this study did not materialize into an exportable clinical protocol or novel device. Steier et al. used a commercially available Neurotrac stimulator to elicit submental stimulation of genioglossus during N2 sleep, with a resolution of upper airway occlusion when activated (58). This work ultimately culminated in the development of the transcutaneous electrical stimulation (TESLA) device. TESLA, a device that utilizes TES, delivers a continuous low-current electrical stimulation to the genioglossus during sleep, which causes increased airway patency. TESLA transmits an electrical current transcutaneously via dermal patches in the sub-mandibular area.

In an RCT, TESLA accounted for multiple positive outcomes. The AHI improved by a mean of 9.1 [95% confidence interval (CI) 2.0, 16.2] events/h, and the 4% oxygen desaturation index (ODI) improved by a mean of 10.0 (95% CI 3.9, 16.0) events/h (51, 59). TESLA exhibited a 100% response rate for mild OSA patients, while patients with moderate and severe OSA reported a 46 and 29% response rate, respectively. While it is still not understood which OSA patients are ideal candidates for TESLA, early studies have identified some features associated with higher success rates. Current inclusion criteria for TESLA include an AHI of 5–35 events/h, a BMI of < 32 kg/m^2^, CPAP intolerance, and low adherence to MAD (60). Adverse effects of TESLA include dry mouth, skin discomfort, and claustrophobia. No major adverse events were reported.

During sleep, the TESLA system included external stimulation of the “upper airway dilators” via 4 x 4 cm patches on the anterior neck. This system appeared to reduce RDI, but which muscles are activated with this program is unclear (59).

There is also the Kalinix device, but limited data have been reported beyond a congress abstract with 20 patients. The authors noted that 52% of individuals had a reduction in AHI, but the exact change is unreported. Inclusion criteria were adults with AHI 15–65 events/h and BMI < 32 kg/m^2^. No serious adverse events were noted. Follow-up studies have not yet been reported (61).

#### Day-time electrical stimulation

Most of the previously mentioned stimulation devices involved transcutaneous stimulation during sleep and included a broad range of OSA severity. Transoral stimulation is a new modality treating mild OSA and simple snoring in individuals with a BMI < 35 kg/m^2^ (62). EXciteOSA, formerly known as Snoozeal, is an oral device that activates the upper airway through electrical stimulation. It includes three components: a control unit, a washable mouthpiece, and a Bluetooth smartphone application. Four electrodes supply the tongue with electrical stimulation. Two electrodes lie on top of the tongue, and two sit below the tongue to generate vertical and diagonal stimulation patterns.

Patients have full control over the intensity of electrical stimulation using their smartphone. The device emits a series of pulse-bursts over 20 min. The frequency of stimulation changes in a defined sequence throughout the treatment cycle. Phase 1 of the treatment includes 20 min once per day, and phase 2 includes 20 min twice per week, though phase 2 of therapy is often individualized in clinical practice.

In the available clinical data, eXciteOSA showed significant improvements in objective and subjective indices of OSA. The AHI reported a mean reduction of 3.4 ± 5.0 events/h (95% CI 2.2–4.7) from 10.2 to 6.8 events/h (*p* < 0.01). The oxygen desaturation index decreased by 2.5 ± 4.6 events/h (95% CI 1.4–3.6) from 8.4 to 5.9 events/h (*p* < 0.01). Mean ESS reduced from 8.7 to 5.3 (reduction of 3.4 ± 4.1; 95% CI 2.4–4.4; *p* < 0.01). Composite Pittsburg sleep quality index (PSQI) decreased from 7.3 to 5.9 (reduction of 1.4 ± 2.8; 95% CI 0.7–2.1; *p* < 0.01). However, further study is needed to identify the optimal patient population for this device. Additionally, it remains unclear how therapy should be modified (if at all) after the initial 6 weeks of treatment. A recent randomized controlled trial has completed enrollment with reportedly favorable results, but the results have not yet been made available to the public. Possible side effects include drooling, tongue tingling, and tooth discomfort (50).

It has been suggested that improving tongue endurance may not influence OSA. In one study evaluating the effects of a six weeklong tongue endurance program, no improvements in OSA severity were detected (63). The exercise regimen did however produce improvements in daytime sleepiness.

### Surgical approaches to upper airway stimulation—hypoglossal nerve stimulation

Hypoglossal Nerve Stimulators (HGNS) are surgically implanted devices that apply electrical stimulation to the hypoglossal nerve to control the movement of the tongue. HGNS is an effective tool to treat OSA because it allows for control of the genioglossus and hence pharyngeal volume. We will include multiple HGNS devices on the market and in development in this review, including the Inspire device, Apnex, Genio, and Aura6000.

### Inspire

Inspire became the only FDA-approved HGNS after the STAR trial in 2014. The initial feasibility study of this model however, dates back to 2001 (64). The Inspire device is surgically implanted into the upper chest, commonly on the right side, and innervates the medial branch of the ipsilateral hypoglossal nerve. Inspire contains three components: a respiratory sensing lead, an impulse generator, and a stimulation lead. The respiratory sensing lead detects the exact phase of the respiratory cycle activating the impulse generator during inspiration. The impulse generator sends an electrical impulse to the hypoglossal nerve through the stimulation lead. Upon electrical stimulation of the hypoglossal nerve, the tongue stiffens and protrudes. Inspire uses both respirophasic and a fixed stimulation pattern. Electrical stimulation strength is modulated with a remote control.

### Benefits and limitations of Inspire

Observational studies have provided some evidence to establish Inspire as a clinically efficacious device in treating OSA (65). In the pivotal STAR trial, HGNS decreased AHI by 68%, from an average of 29.3 events per hour to 9.0 events per hour (65). The ODI score decreased by 70%, from 25.4 events per hour to 7.4 events per hour (65). Secondary outcomes, including the Functional Outcomes of Sleep Questionnaire (FOSQ) and ESS, also showed improvement (65). This trial was followed by a therapy-withdrawal study which randomly assigned responders to withhold HGNS temporarily. Results from this study showed responders taken off HGNS returned to baseline in both AHI and ODI. When HGNS was re-initiated, the AHI and ODI returned to post-treatment standards (65). The most comprehensive data set on HGNS is the ADHERE Registry, which includes patient-level data for individuals who have undergone HGNS. Analysis of this data set further confirms the significant therapeutic effects of HGNS on both objective and subjective measures of OSA (66). This registry now includes nearly 5,000 patients with longitudinal data.

Patient selection for Inspire is based on criteria informed by the STAR trial (65). Indications for implantation include moderate to severe OSA with CPAP intolerance or refusal. Patients must have a BMI < 32 kg/m^2^; < 25% central/mixed apnea events, and an AHI between 15 to 65/h (65). Contraindications for HGNS include a complete concentric collapse of the soft palate (65). Candidacy requirements for HGNS devices are still evolving.

HGNS appears well-tolerated, but 1/3 of patients have been deemed non-responders long-term (66). To optimize patient selection for HGNS, Op de Beek examined OSA endotypes and noted that those with a higher arousal threshold, greater muscle compensation, and lower loop gain had a higher chance of HGNS success (67). Conversely, patients with low muscle compensation and mild collapsibility were noted to have lower HGNS success rates (67). Additionally, higher baseline AHI, lower BMI, and older patient age appear to be associated with a greater reduction in AHI with HGNS (68). These results suggest diagnosing the non-anatomical characteristics of OSA may play a critical role in prescribing HGNS (67).

From an anatomic perspective however, complete palatal and complete tongue base collapse, but not complete lateral pharyngeal wall collapse as assessed by drug-induced sleep endoscopy (DISE) are associated with greater AHI reduction following HGNS implantation (69). Additionally, tongue morphology during stimulation is important for maintaining airway patency (70). Tongue protrusion and maintenance of tongue shape is associated with increased airflow, whereas anterior movement with increases in tongue height are associated with decreased airway patency (70). Lastly, both the extrinsic and intrinsic muscles of the tongue appear to be activated by HGNS, with the milieu of muscles activated depending on cuff position, voltage intensity, and pattern of stimulation (71). Thus, there is tremendous complexity underpinning patient selection, therapy optimization, and non-anatomic traits in generating an optimum response to HGNS.

### Apnex

One of the initial HGNS device studied was the Apnex device (72). This device has a single stimulation lead and two respiratory sensing leads (73). Cuff placement is on the main branch of the hypoglossal nerve, distal to the branches innervating tongue retractors (determined intraoperatively through stimulation). This device was reported to be well-tolerated and significantly reduced AHI, particularly in those with a BMI < 35 kg/m^2^ (73). This device, however, is no longer actively studied and is not clinically available.

### Genio

Genio is a bilateral HGNS device produced by Nyxoah (74). Genio provides stimulation to both branches of the hypoglossal nerve (65). This device requires a single midline submental incision with placement of paddled electrodes over bilateral distal medial hypoglossal nerve branches. The preferential selection of the distal branches reportedly activates genioglossus alone without the recruitment of adjacent muscles (74). An external, submental stimulator is placed on an adhesive, disposable patch to activate the cuffs (74). The stimulator must be recharged daily but has the advantage of not having an implanted battery. The Genio does not have respiratory sensing leads and delivers stimulation via adjustable, pre-programmed rates and duty-cycles in order to match the patient's breathing frequency. The BLAST OSA study was pivotal for this device (74). Inclusion criteria were adults 21–65 years old, AHI 15–65 events/h, BMI < 32 kg/m^2^, and fewer than ten central events/h on PSG (74). This study did not meet its primary endpoint of an AHI reduction of 15 events/h, but AHI was significantly reduced from 23.7 ± 12.2 to 12.9 ± 10.1 and ESS from 11.0 ± 5.3 to 8.0 ± 5.4 (74). Quality of life metrics and bed-partner-reported snoring were also considerably reduced. No serious adverse events were reported (74).

In a study comparing unilateral HGNS and bilateral HGNS, no significant differences were detected in the AHI or ESS between the two treatment groups (75). This evidence suggests bilateral HGNS may be as a safe and effective as unilateral HGNS.

### Aura6000

The Aura6000 is an emerging technology from LivaNova (previously under ImThera). The Aura6000 does not have a respiratory sensing component and assessment for concentric collapse by DISE is not part of the clinical workflow (76). The Aura6000 electrodes are placed in an unfasciculated portion of the hypoglossal nerve, targeting multiple muscle groups in the fatigue-resistant components of the posterior tongue (77). The rate of serious adverse events appears to be comparable to Inspire (25). The inclusion criteria for ongoing studies include adults over 22 with AHI 20–65/h and CPAP refusal or intolerance. Exclusion criteria include BMI > 35 kg/m^2^, comorbid pulmonary, cardiac, or renal disease, and detailed PSG exclusion criteria, most notably, the presence of central or mixed apneas in >25% of AHI events (78). Based on the recent THN3 trial, data at 12–15 months for enrolled participants suggest that AHI is reduced by 42.5% percent within their cohort (25).

### Ansa cervicalis stimulation

Stimulation of the ansa cervicalis as a therapeutic target to treat OSA can be used alone or in combination with HGNS (79). The ansa cervicalis is a nerve plexus innervating the infrahyoid strap muscles including the sternothyroid muscle. When activated, these muscles create caudal displacement of the hyoid bone, resulting in a stiffened upper airway (80, 81). In a small clinical study, stimulation of the ansa cervicalis increased inspiratory airflow in patients with severe OSA during DISE (79). Ansa Cervicalis Stimulation (ACS) increases pharyngeal volume by increasing caudal traction of the upper airway (82).

ACS may help overcome incomplete responses to HGNS (83). The combined effect of tongue protrusion and tracheal traction is likely synergistic (80). Early data on ACS are limited by small sample size, low diversity of study population, and lack of data accounting for end-expiratory lung volume (79). However, it has been shown that ACS decreases PCrit and Popen (when nasal pressure exceeds surrounding tissue pressure), with a significantly greater improvement in Popen with bilateral vs. unilateral ACS (84). It is challenging to compare HGNS and ACS due to different stimulation patterns. Despite these limitations, ACS has shown robust improvements in airway collapsibility and should be further investigated.

### Summary and future directions

There is a rich history of improving upper airway output as a therapeutic modality in OSA (52, 78). Efforts have included MT, woodwind instruments, external stimulation devices, and direct nerve stimulation of varying regions of the hypoglossal nerve and the ansa cervicalis. A comprehensive consensus statement on non-PAP therapies was issued by the European Respiratory Society in 2021. Notably, the quality of evidence for many non-PAP interventions appears to be poor (43). Each intervention has improved OSA with routine use, but it is unclear which patients and endotypes benefit from each modality (85). We anticipate that the future of OSA therapy will include tailoring interventions to OSA traits and patient preferences, which will allow for optimum therapeutic engagement. Despite being a relatively young field, with < 50 years of history, tremendous progress has been made in the application of bench physiology to the bedside in the management of OSA. Improving upper airway mechanics is just one approach, but considerable nuance is involved in a task as seemingly simple as stabilizing the pharyngeal airway. Similar granularity is required in addressing the other endotypes as well. As such, the future of our field includes precision medicine toward unique combinations of endotypic traits, multiple lines of concurrent therapies, and therapeutic adjustments as individual patient physiology evolves (13). We view this challenge with great excitement and believe tremendous opportunities to individualize patient care in OSA lie ahead.

## Author contributions

All authors contributed substantially to the development of the manuscript, its editing, and final manuscript preparation.
